# Tuning spacer length improves the functionality of the nanobody-based VEGFR2 CAR T cell

**DOI:** 10.1186/s12896-023-00827-0

**Published:** 2024-01-04

**Authors:** Fatemeh Hajari Taheri, Mahmoud Hassani, Zahra Sharifzadeh, Mahdi Behdani, Shahryar Abdoli, Mahtab Sayadi, Kowsar Bagherzadeh, Arash Arashkia, Mohsen Abolhassani

**Affiliations:** 1 Hybridoma Lab, Department of Immunology, Pasteur Institute of Iran, Tehran, Iran; 2 Food and Drug Laboratory Research Center (FDLRC), Iran Food and Drug Administration (IFDA), MOH & ME, Tehran, Iran; 3 Medical Nanotechnology and Tissue Engineering Research Center, Shahid Beheshti University of Medical Sciences, Tehran, Iran; 4 Department of Medical Biotechnology, School of Advanced Technologies in Medicine, Shahid Beheshti University of Medical Sciences, Tehran, Iran; 5 Department of Medical Biotechnology, Biotechnology Research Center, Pasteur Institute of Iran, Tehran, Iran; 6 Department of Medical Biotechnology, Golestan University of Medical Science, Gorgān, Iran; 7 Cellular and Molecular Research Center, Birjand University of Medical Sciences, Birjand, Iran; 8 Eye Research Center, Five Senses Health Institute, Rassoul Akram Hospital, Iran University of Medical Sciences, Tehran, Iran; 9 Department of Molecular Virology, Pasteur Institute of Iran, Tehran, Iran

**Keywords:** Chimeric antigen receptor (CAR), IgG1 CH2-CH3, Hinge, Spacer domain, VEGFR2

## Abstract

**Background:**

The chimeric antigen receptor-expressing T (CAR-T) cells for cancer immunotherapy have obtained considerable clinical importance. CAR T cells need an optimized intracellular signaling domain to get appropriately activated and also for the proper antigen recognition, the length and composition of the extracellular spacer are critical factors.

**Results:**

We constructed two third-generation nanobody-based VEGFR2-CARs containing either IgG1 hinge-CH2-CH3 region or hinge-only as long or short extracellular spacers, respectively. Both CARs also contained intracellular activating domains of CD28, OX40, and CD3ζ. The T cells from healthy individuals were transduced efficiently with the two CARs, and showed increased secretion of IL-2 and IFN-γ cytokines, and also CD69 and CD25 activation markers along with cytolytic activity after encountering VEGFR2^+^ cells. The VEGFR2-CAR T cells harboring the long spacer showed higher cytokine release and CD69 and CD25 expression in addition to a more efficient cytolytic effect on VEGFR2^+^ target cells.

**Conclusions:**

The results demonstrated that the third-generation anti-VEGFR2 nanobody-based CAR T cell with a long spacer had a superior function and potentially could be a better candidate for solid tumor treatment.

**Supplementary Information:**

The online version contains supplementary material available at 10.1186/s12896-023-00827-0.

## Background

Vascular abnormalities are one of the hallmarks of solid tumors, and the vascular endothelial growth factor (VEGF) family plays a leading role in their induction. VEGFR2, the primary receptor for VEGF-A, is overexpressed in many metastatic cancers, and its signaling is involved in tumor cell proliferation, migration, and invasion [1, 2]. Therefore, VEGFR2 has emerged as an attractive target for adoptive cancer immunotherapy.

In recent years, one of the promising approaches to treat cancer has been the introduction of CARs into the T-cells to redirect their antigen specificity and immune function [3–5]. The CARs typically have four distinct regions, including an extracellular section responsible for target antigen binding, a hinge or spacer that separates the binding moieties from the transmembrane section, a transmembrane section that anchors the CAR in the cell membrane and also is involved in T-cell function and an intracellular region containing costimulatory domains that are linked in cis position and mediate cell signaling [6–8].

So far, five generations of CARs have been described. First-generation CARs contained an intracellular CD3ζ-signaling domain and were not able to prime resting T cells and direct the T-cell responses due to their limited signaling capability [9]. In the second- and third-generation CARs, one and two more costimulatory signaling domains (CD28, 4-1BB, and OX40), respectively, were utilized to improve activation, survival, and effective expansion of the T cells [10]. In fourth- and fifth-generation CARs, the ability of antitumor function was further enhanced by new genetic modifications for the expression of transgenic proteins such as cytokines and an additional membrane receptor such as cytokine receptors, respectively [11, 12].

One of the critical parts for developing a functional CAR T-cell is the spacer between the extracellular antibody and transmembrane section, which its length and composition can affect CAR expression, flexibility, epitope recognition, and signaling [13–15]. Although optimal spacer length depends on factors such as position and density of the ligand, the proper spacer length may have to be tailored for each specific epitope. Spacer domains of the CARs have been mainly adopted from flexible regions of CD28, CD8α, and, more commonly, the Fc region of IgG1 and IgG4 antibodies. Nevertheless, IgG1 Fc spacer domain can result in the ligand-independent activation through binding to FcγR-expressing immune cells. Several amino acid sequences are present within the IgG1 Fc CH2 domain that can be recognized and bound by Fc receptors, and replacing some of them have shown to prevent their attachment to FcγR [16].

Previously, we constructed a second-generation camelid VHH-harboring CAR to target VEGFR2-positive tumor cells [17]. VHHs are the most miniature antibodies comprised of a single-domain and have a high homology to the human VH sequence [12]. In this report, we developed two new third-generation CARs using two different lengths of extracellular spacer domains derived from the Fc region of IgG1 to evaluate their efficiency in recognition of VEGFR2-expressing tumor cells in vitro. We used OX40 as the second costimulatory signaling domain due to its promising potentiality for enhancing the persistence and reducing the exhaustion of CAR T cells in addition to the metabolic advantages associated with OX40 signaling [18]. We finally showed that the longer spacer had considerably affected the third-generation anti-VEGFR2 CAR effector functions.

## Results

### Transduction and expression of CARs in T cells

**Fig. 1:**
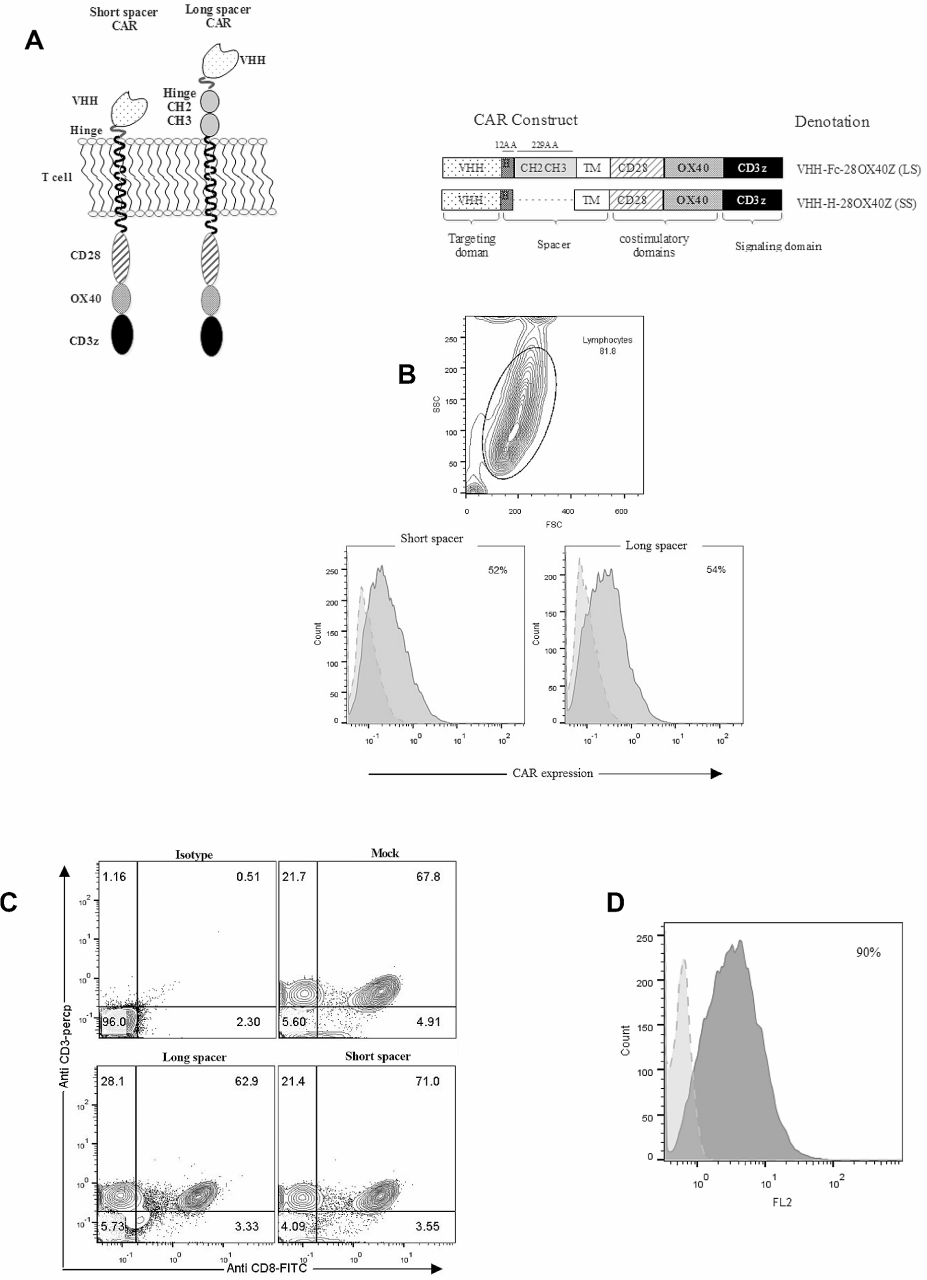
Q1Analysis of CAR and VEGFR-2 expression on the cell surface. (A) Scheme of the short and long spacer CARs used in this study and the positioning of their coding segments. (B) Analysis of CAR expression on the surface of T cells from one of the donors transduced with vectors encoding the VEGFR2-CARs with the expression of the short spacer CAR on 52% and long spacer CAR on 54% of the T cells, while undetectable on mock-transduced T cells (light grey histogram). (C) Phenotypic analysis of the T cells seven days post transduction. The CD3 + T cells were 91.4 ± 3.4% of the T cell population and the majority of them (67 ± 5.3%) consisted of CD8 + T cells. The results of Isotype control, along with mock, long spacer and short spacer CAR transduced T cells for on donor have been represented. (D) Studying VEGFR2 presentation on the surface of 293-KDR and HEK-293 cells using polyclonal antibodies. The deep grey histogram shows 293-KDR (90%), and the dotted histogram represents HEK-293

We constructed two lentiviral vectors encoding third-generation nanobody-based CARs with either IgG1 hinge-CH2-CH3 (229 aa) long or hinge (12 aa) short spacers against VEGFR2. In both constructs, the extracellular segments were linked to the intracellular CD28, OX40, and CD3ζ motifs via a CD28 transmembrane domain (Fig. 1A). The T cells’ transduction efficiency was 45–55% using the second**-**generation lentiviral vector system. The cell surface expression of VEGFR2-CAR was detected through staining the transduced T cells. The results for one of the donors representatively showed that 52% of transduced T cells expressed the short spacer (SS) CAR, and 54% of them expressed the long spacer (LS) CAR. To serve as a negative control, mock-transduced T cells were used (Fig. 1B). Also, analysis of the T-cell phenotype at seven days post-transduction showed that 91.4 ± 3.4% of the T cell population was CD3^+^, and among them, 67 ± 5.3% were CD8^+^ (Fig. 1C). The surface expression of VEGFR2 on 293-KDR and HEK-293 cells was analyzed using specific polyclonal antibodies (Fig. 1D).

### CAR-induced cytokine secretion

**Fig. 2:**
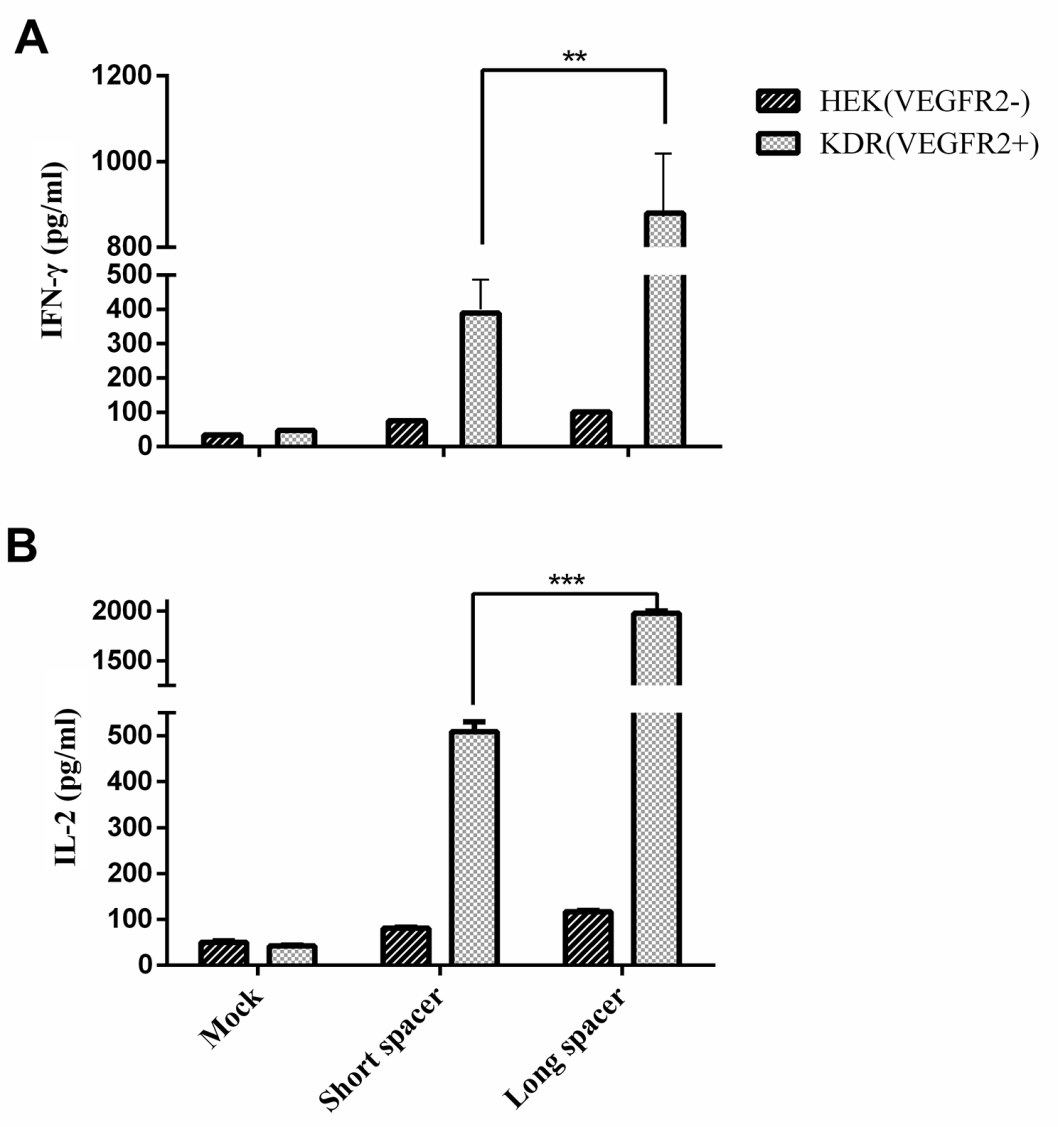
Production of IFN-γ (**A**) and IL-2 (**B**) as the indicator of CAR T cell activation in response to antigen stimulation. CAR T cells or mock-transduced T cells were co-cultured with either VEGFR2-expressing 293-KDR or VEGFR2^−^ HEK-293 cell lines, and the cytokine level in the co-culture medium was measured using ELISA. The concentrations of IFN-γ and IL-2 were 879 pg/ml and 1924 pg/ml, respectively, in the case of long-spacer CARs and 369 pg/ml and 510 pg/ml for short-spacer VEGFR2-CAR T cells. Results were presented as means ± SD of duplicate assays (n = 3) (***p < 0.001, **p < 0.01)

We measured IFN-γ (Fig. 2A) and IL-2 (Fig. 2B) secretion as the indicators of CAR T cell activation in response to antigen stimulation. A marked secretion of both IFN-γ and IL-2 was observed in response to co-culturing with 293-KDR cells compared to the levels detected in response to the HEK-293 cells. Stimulation of VEGFR2-CAR T cells expressing long spacer resulted in secretion of 879 ± 18.5 pg/ml and 1924 ± 18.4 pg/ml of IFN-γ and IL-2, respectively, which were significantly higher than the concentration of the same cytokines in case of VEGFR2-CAR T cells expressing short spacer that were 369 ± 25.5 pg/ml and 510 ± 17.6 pg/ml, respectively. Our results demonstrated that VEGFR2-CAR T cells, in comparison to the mock group, had a statistically significant higher level of cytokine secretion (42 ± 1.6 pg/ml and 61 ± 4.9 pg/ml of IFN-γ and IL-2) (*p* < 0.001).

### Activation markers surface expression on VEGFR2-CAR T cells

**Fig. 3:**
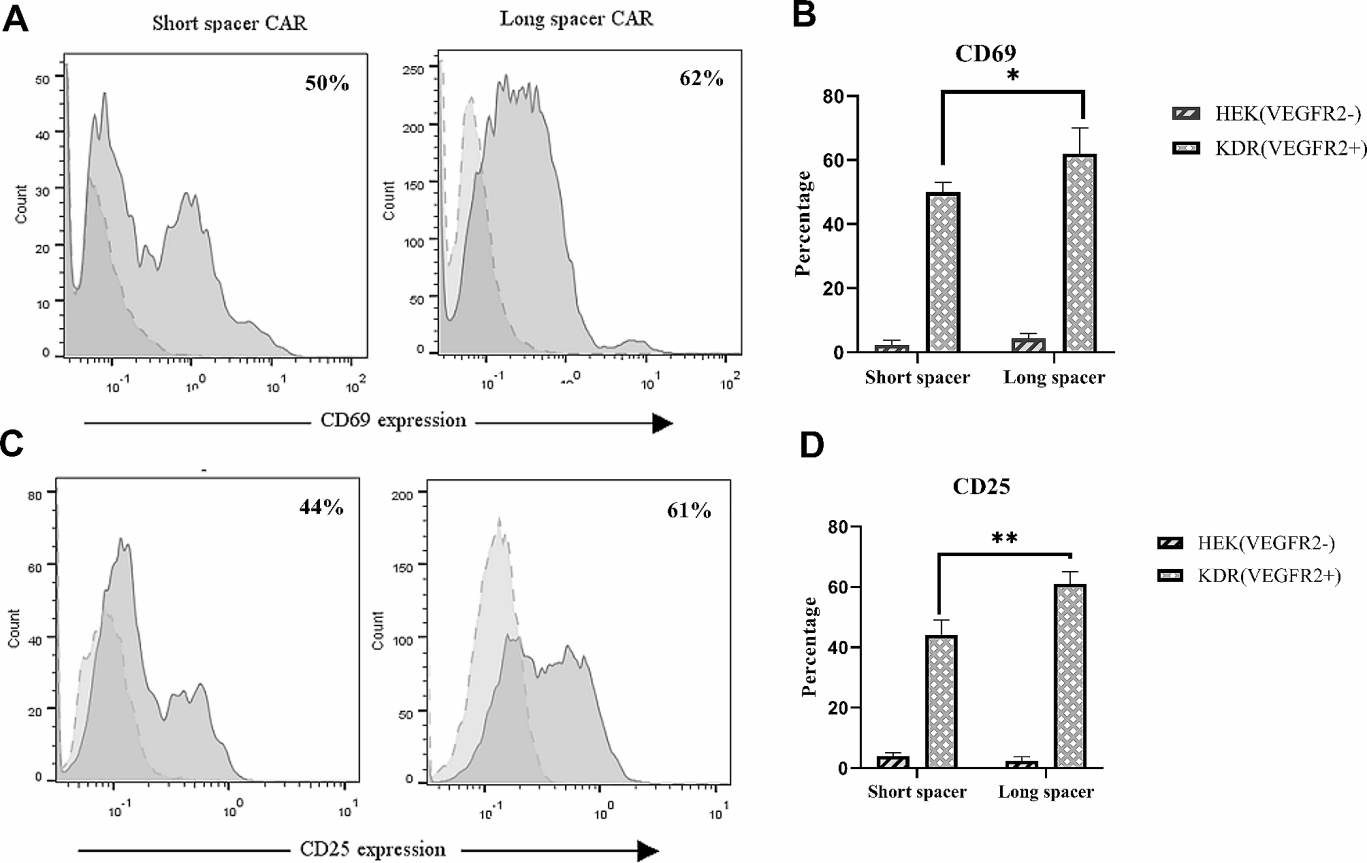
Expression of CD69 (A and B) and CD25 (C and D) in VEGFR2-CARs with long and short extracellular spacers. T cells modified with the VEGFR2-CARs expressed CD69 and CD25 when co-cultured with 293-KDR cells but not with HEK-293 cells (dotted histograms). Long spacer CAR T cells expressed higher levels of CD69 (62 ± 6.5%) and CD25 (61 ± 3.3%), compared with the short spacer CAR-expressing CD69 (50 ± 2.4%) and CD25 (44 ± 4.1%). Results were presented as means ± SD of duplicate assays (n = 3) (**p < 0.01, *p < 0.05)

Lymphocytes stimulation led to the upregulation of cell surface markers at various phases of cellular activation. CD69 (Fig. 3A and B) and CD25 (Fig. 3C and D) were regarded as very early and late T-cell activation markers, respectively. As shown in Fig. 3, T cells modified with the VEGFR2-CARs were efficiently activated when co-cultured with 293-KDR cells but not with HEK-293 cells (dotted histograms. Long spacer CAR T cells expressed higher levels of CD69 (62 ± 6.5%) and CD25 (61 ± 3.3%) as compared with the short spacer CAR-expressing cells (50 ± 2.4% and 44 ± 4.1%, respectively).

### Cytotoxicity of CAR T cells upon co-culturing with the target cells

**Fig. 4:**
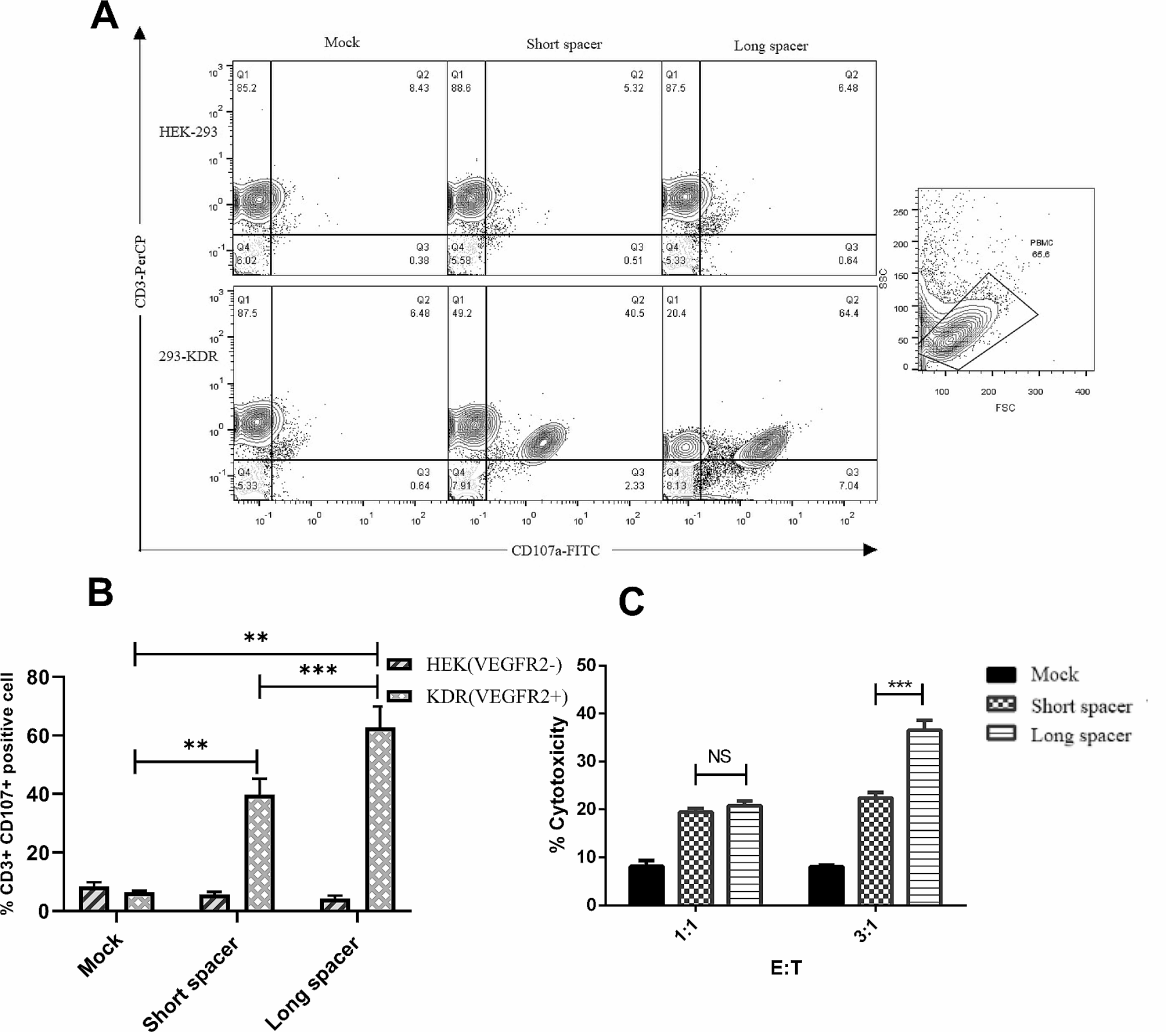
Cytolytic activity of VEGFR2-CAR T cells with either a long or a short extracellular spacer domain. (A) The panel shows CD107a expression on CD3 + T cells from a representative donor transduced with mock, and long- and short-spacer-containing CAR constructs. (B) Data from two individual donors in two different experimental environments. The CD107a expression was significantly higher in the case of long spacer CAR (64.4 ± 3.8%) compared with short spacer CAR (40.5 ± 3.3%), p < 0.05. (C) CFSE/PI cytotoxicity assay of short and long spacer VEGFR2-CAR T cells at E:T ratios of 3:1 (35 ± 3.2% vs. 21.5 ± 2.4%, respectively) and 1:1 (both 20%) after co-culturing of CAR T cells with VEGFR2 + cells (***p < 0.001, **p < 0.01)

To analyze the cytotoxicity function, CD107a expression on the T cell surface, as a degranulation marker, was measured four hours after co-culturing with VEGFR2-expressing target cells. The results showed CD107a expression on the VEGFR2-CAR T cells was 64.4 ± 3.8% for the long spacer and 40.5 ± 3.3% for the short spacer (Fig. 4A and B). CFSE-PI labeling was used for assessing the potency of target cell lysis, and the results showed that both short and long spacer-harboring CAR T cells could lyse the 293-KDR cells in 1:1 and 3:1 E:T ratios. Additionally, the VEGFR2-CAR T cells expressing long spacer had significantly more cytotoxic activity than the short spacer-containing counterpart in 3:1 E:T ratio (35 ± 3.2% for long spacer, and 21.5 ± 2.4% for short spacer) (Fig. 4C) that was in accordance with the CD107a expression results.

## Discussion

We previously showed that the second-generation VEGFR2-CAR T cells with long spacer could be activated by target cells expressing VEGFR2 molecule, and produce Th1 cytokines and kill the target cells [17]. In this study, we developed two third-generation CAR T cells containing human IgG1 Fc-based spacers to evaluate the effect of spacer length on the activation of VEGFR2-CAR T cells. Following confirming that the surface expression of the two VHH-based CARs was similar, their activity was assessed in vitro against VEGFR-2 expressing 293-KDR cell line. Our data showed that antigen recognition by CAR T cells containing long spacer domain resulted in more efficient expression of CD25 and CD69 activation markers in addition to IFN-γ and IL-2 production, as compared with short spacer CAR T cells. Additionally, expression of CD107a (LAMP1) degranulation marker and cytolytic activity were significantly higher in case of the long spacer.

The hinge-CH2-CH3 segment of IgG Fc is one of the most widely used spacers in CAR constructs [14, 19–24] due to its low immunogenicity, easy detectability by anti-Fc antibodies, and the potentiality for removing and substituting its domains to provide optimal spacer length and composition [23]. It has been proposed that the optimal spacer length depends on both the accessibility and also the location of the targeted epitope to ensure a suitable intercellular distance between the CAR-T and target cells [25]. Accordingly, a previous report about CD22-specific CAR showed that the distance between the antigenic epitope and the cell membrane of the effector cells was significant [26]. Tumor recognition by MUC1, 5T4, and NCAM-specific CARs was more effective when the long spacer was used (longer/flexible) [14, 27]. Studying the HER-2/neu (ERBB2) receptor, optimum T-cell activation was only observed when the spacer domain incorporated into the CAR consisted of the hinge regions [15]. L1CAM short spacer-CD28/ζ CAR T cells expanded and induced initial tumor regression more than the long spacer at the tumor site in the face of the cancerous target [28]. Supposedly, in case of close proximity of the epitope to the cell membrane, a short spacer region in the CAR receptor may not provide sufficient spacing to allow optimum T-cell activation, and when the target epitope is distant from the cell surface (e.g., MFE23), a long spacer may not be required [29, 30]. On the contrary, when the target epitope has close proximity to the cell membrane (as with the epitopes of NCAM, 5T4, and MUC1), short spacer regions, as were applied in the anti-NCAM and anti-5T4 receptors, can lead to deficient T-cell activity [14]. Chang et al. explained the T cell activation and distance between the T cell and cognate antigen by the kinetic segregation model [31]. Accordingly, when T cells contacted the antigen, an immunological synapse would be formed that could exclude big CD148 or CD45 phosphatases, which had an inhibitory function; otherwise, the inhibitory phosphatases could abort T cell activation by entering the synapse [31, 32]. These data suggest that for every target antigen, the length of the extracellular spacer should be modified. Generally, membrane-distal epitopes can activate short spacer CARs most efficiently, while long spacer CARs interaction with membrane-proximal epitopes can lead to efficient elicitation of the CAR T cell, depicting the fundamental role of the optimum distance between CAR T and the target cells [14, 29].

Our preliminary in silico analyses showed that the anti-VEGFR2 nanobody could bind to a distal epitope (Fig. S1), but the in vitro tests showed the use of a long spacer CAR induced a higher CAR T cell activity. Therefore, merely knowing the location of the epitope in the membrane, whether it is distal or proximal, is insufficient to determine the optimal spacer length. In vitro testing is necessary to determine the optimal spacer length that provides maximum efficiency for the CAR. The distance between the target cell and the T cell in addition to the location of the target epitope are two crucial factors that must be considered in CAR design. These factors may affect tumor recognition, T cell signaling, and synapse formation between the tumor cell and the T cell [33]. These findings indicate the necessity of studying various constructs for each particular CAR/epitope interaction to find optimum spacer length that can result in efficient chimeric receptor activity.

Although the Fc region of IgG is one of the most common spacers used for optimizing immunologic synapse in CAR designing, the ability of its CH2 domain to bind FcγR-harboring NK cells and monocytes is considered a kind of limitation [28, 34]. CH2- FcγR interaction can elicit cells of the innate immune system and limit CAR-bearing cell persistence, in addition to the increased activation-induced cell death. Removing the CH2 and/or CH3 domains in addition to substituting essential amino acids in the CH2 domain that take part in binding to the FcγR can lead to the disruption of this undesirable interaction. This consideration is more critical for optimal antigen binding when designing long spacer CARs [35, 36].

The scFvs have two single domain antigen-binding modules which are connected with a linker, and they have reportedly strong tendency for self-aggregation. Accordingly, the CARs’ interactions within the scFv network can elicit tonic signaling in engineered effector cells, and extreme tonic signaling in an antigen-independent pathway may finally cause early exhaustion of engineered effector cells [37]. Given that VHHs have a single antigen-binding domain, they do not interfere with one another, and there is no tonic signaling in VHH-based CAR. This issue was more evident in our results considering that upon co-culturing of CAR T cells with VEGFR2^−^ HEK-293 cells there were neither CD69/CD25/CD107 upregulation nor a significantly higher cytokine production.

We previously showed that a second-generation CAR harboring only the CD28 domain was functional. Several studies have shown that in vitro and in vivo functions of CARs containing one (second generation) or two (third generation) costimulatory domains are superior to that of first-generation CARs that only contained CD3ζ signaling domain [38–42]. In the present study, we used third-generation CAR by adding an extra OX40 costimulatory domain. The OX40 elicits a wide range of T-cell responses, such as proliferation and differentiation, cytokine and chemokine secretion, cytolytic activity, and protection from activation-induced cell death [43]. Compared to our previously developed second generation CAR, the present CAR resulted in more CD69 expression as the very early T cell activation marker [44]. A study by Hombach et al., showed that OX40 co-signaling in a third-generation CD28-ζ-OX40 CAR repressed CD28-mediated IL-10 production but did not affect the production of pro-inflammatory cytokines, T-cell proliferation, and T-cell mediated cytotoxicity [45]. Mestas et al. have used OX40L knockout or transgenic mice and showed that OX40 had a role in immune response regulation [46]. Although OX40 did not change the IL-2 transcription, it may increase its half-life by 3–6 folds and, therefore could stabilize a subset of IL-2 mRNA [46].

In the previous study, we used plasmid DNA electroporation for CAR gene transfer, which resulted in low CAR expression and also required re-stimulation and antibiotic selection of transfected cells. In this study, we efficiently transduced T cells with the second-generation lentiviral vector, and the functionality of CAR T cells were analyzed in vitro. However, in vivo analyses are needed to demonstrate the potentiality of the currently developed CAR T cells to effectively target the tumor along with T cell expansion and generation of memory T cells.

## Conclusions

In summary, we redirected human T cells by two nanobody-based VEGFR2-specific CARs containing either long or short spacers. The CAR T cells were potently activated following co-culturing with VEGFR2^+^ cells in vitro, and the values of the activation parameters in the case of long spacer-containing CAR T cells were significantly higher than the short spacer CAR T cells, making them more appropriate candidates for further in vivo studies.

## Methods

### Designing the CAR constructs

The CAR constructs of this study have been shown schematically in Fig. 1A. The various domains in different CAR constructs have been ordered as follows: The VHH-Fc-28OX40Z CAR included a VHH against human VEGFR-2 [47] that was linked in-frame to either a human IgG1 hinge-CH2-CH3 domain as the long spacer (LS) or only an IgG1 hinge as the short spacer (SS). Based on the Hombach et al. study, we modified the IgG1 Fc spacer in the CH2 domain by applying two L235D and N297Q substitution mutations in the CH2 domain that had been demonstrated to inhibit activation-induced cell death through FcγR interaction [35]. The human CD28 and OX40 were used as costimulatory domains, and the human CD3ζ was incorporated as the intracellular signaling domain. The pCDH lentiviral vector (Bioscience, USA) was used to sub-clone the constructs that were named VHH-Fc-28OX40Z (LS) and VHH-H-28OX40Z (SS) CARs. The empty vector of pCDH was used as the mock control.

### Packaging and transduction of VEGFR2 CAR-encoding lentiviral vectors

To generate lentiviral vectors, 8 × 10^6^ Lenti-X 293T cells (Clontech Laboratories, USA) were cultured in a 10 cm^2^ plate in Dulbecco’s Modified Eagle Medium (DMEM) (Gibco, USA) supplemented with 10% fetal bovine serum (FBS), 100 µg/mL streptomycin, and 100 unit/mL penicillin, 24 h prior to transfection, and incubated at 37 ºC in a humidified 5% CO_2_ chamber. The second-generation lentivirus packaging system contained psPAX and pMD2.G packaging plasmids along with pCDH-CAR constructs that were co-transfected into the Lenti-X 293 cells with Lipofectamine 3000 transfection reagent (ThermoFisher, USA) according to the provider’s instructions. Virus-containing supernatant was collected every 24 h for three days. The collected supernatants were centrifuged for 90 min at 50,000 × g at 4 °C, followed by filter sterilization and storage at -80 °C for further use.

### Primary T cells activation and transduction

We attempted to isolate T cells from five different healthy donors. Unfortunately, technical issues including CO_2_ incubator malfunction and bacterial contamination in the cell cultures led to the loss of three samples. As a result, we proceeded with T cells from only two healthy donors (informed consent was obtained from all cases) that were stimulated by applying 10 µg/mL soluble anti-CD3 mAb OKT3 (BioLegend, USA) and 1 µg/mL soluble anti-CD28 mAb 15E8 (BioLegend, USA) in the presence of 20 IU/ml rhIL-2 (R&D System, UK) for 24 h. The study was approved by the Ethics Committee in Pasteur Institute of Iran (IR.PII.REC.1398.009). The CAR-encoding lentiviruses at MOI 5 along with a final concentration of 8 µg/ml polybrene (Sigma, USA) were added to the stimulated T cells and cultured in 6 cm^2^ dishes. Cells were centrifuged for 90 min at 400 × g at 32 °C and were kept for 24 h. The transduction was repeated one more time, and the culture medium was changed every 48 h, and rhIL-2 (20 IU/mL) (R&D System, UK) was routinely added.

### Detecting the cell surface expression of CAR and VEGFR2

Expression of long and short spacer CARs on human T cells was measured by indirect immunofluorescence using recombinant murine VEGFR-2–hIgG-Fc fusion protein (R&D Systems, UK) as the CAR-binding Ag and staining with a FITC-labeled goat anti-mouse (hIgG-Fc) antibody (Cat # A16085, Life Technology, USA).

In this study we used 293-KDR cell line, a modified version of the HEK-293, as the VEGFR2-expressing cell line that has been used in previous studies [17, 48–50] and in contrary to human umbilical vein endothelial cell (HUVEC), is a high VEGFR2 expressing cell line (expressing 2.5 × 10^6^ VEGFR2 per cell) [51]. Expression of VEGFR-2 on human HEK-293 (VEGFR2^−^) and 293-KDR (VEGFR2^+^) was detected using murine anti-human VEGFR-2-PE antibody (R&D Systems, UK), based on the manufacturer protocol. All cell lines were routinely analyzed to confirm the expression of the expected surface markers by flow cytometry.

### Analysis of cytokine production

A population of 6 × 10^4^ transduced T cells were co-cultured with 2 × 10^4^ target cells (HEK-293 and 293-KDR). After 24 h, the supernatant was collected, and the concentration of IFN-γ and interleukin (IL)-2 were measured by a commercial ELISA kit (R&D Systems, UK) based on the manufacturer guidelines.

### Immunophenotyping of CAR T cells

The cell surface expression of CD69 and CD25 on the activated human T cells was analyzed by flow cytometry using conjugated corresponding antibodies (anti-human CD69 antibody Cat# 310,902 and anti-human CD25 antibody Cat# 311,702, BioLegend, USA). The VEGFR2-CAR T cells and mock-transduced T cells were co-cultured with HEK-293 and 293-KDR cells at an E:T (effector to target cell) ratio of 1:1. After 24 h, the expression of CD69 and 48 h later, the expression of CD25 were detected by flow cytometry [52, 53]. The expression of CD8 on the surface of activated human T cells was evaluated by flow cytometry using anti-CD8 (Cat# 344,702) antibody (BioLegend, USA) followed by staining with conjugated goat anti-mouse secondary antibody (Cat#405,305, BioLegend, USA). VEGFR2-CAR and Mock- transduced T cells (10^5^ cells) were co-cultured with 293-KDR and HEK-293 cells at 1:1 and 3:1 E:T ratios in 96-well plates for 4 h. The cells were then stained using the PE-conjugated anti-CD107a antibody (Cat# 328,607, BioLegend, USA) and PerCP-conjugated anti-CD3 antibody (Cat# 344,813, BioLegend, USA), and after 5 hours were analyzed with flow cytometry.

### Cytotoxicity assay

The 293-KDR and HEK-293 cells were labeled with 0.2 µM CFSE (eBioscience, USA). The CFSE-labeled target cells were then washed and co-cultured with 2 × 10^5^ transduced T cells and Mock-transduced effector T cells at E:T ratios of 3:1 and 1:1 for 24 h, followed by target cell viability assay using propidium iodide (PI, eBioscience, USA) exclusion/flow cytometry. According to the Finney study, the target cell lysis percentage was calculated as: (percentage of viable target cells in the absence of effector cells) - (percentage of viable target cells in the presence of effector cells) [7].

### Statistical analysis

Statistical tests for IFN-γ and IL-2 secretion and CD107a expression were performed using the paired *t*-test, and the analysis for cell viability using propidium iodide staining was performed using two-way ANOVA. The analyses were conducted using GraphPad Prism version 7 (La Jolla, California, USA), and values of *p* < 0.05 were considered to be statistically significant.

### Electronic supplementary material

Below is the link to the electronic supplementary material.

Supplementary Material 1

## Data Availability

All authors declare that the data generated or analyzed during this study are included in this published article.

## Abbreviations

CAR-T cell: The chimeric antigen receptor-expressing T cell
VEGF: vascular endothelial growth factor
VEGFR2: vascular endothelial growth factor receptor 2
CD28: Cluster of Differentiation 28
ELISA: enzyme-linked immunosorbent assay
CD69: Cluster of Differentiation 69
CD25: Cluster of Differentiation 25
IFN-γ: Interferon‐gamma
IgG: Immunoglobulin G
CD107a: Cluster of Differentiation 107 alpha
IL-2: interleukin-2
IFN‐γ: Interferon‐gamma
FcγR: Fc gamma receptor
KDR: Kinase insert domain receptor
HEK: Human Embryonic Kidney
VHH: variable domain of heavy chain antibody
CFSE: Carboxyfluorescein diacetate, succinimidyl ester
PI: propidium iodide
PerCP: Peridinin-chlorophyll-protein complex
