# Supplementary material for: Tuning spacer length improves the functionality of the nanobody-based VEGFR2 CAR T cell
Source: BMC Biotechnol. 2024 Jan 4;24:1. doi: 10.1186/s12896-023-00827-0 (PMC10768260; doi:10.1186/s12896-023-00827-0)
Supplement: Supplementary file 1 — Supplementary Material 1 [file 12896_2023_827_MOESM1_ESM.docx]

**In silico binding prediction of the nanobody on VEGFR2**

Vascular endothelial growth factor receptor 2 FASTA sequence was retrieved from Uniprot (P35968·VGFR2_HUMAN). The SWISS-MODEL workspace (https://swissmodel.expasy.org/) was used to homology model VGFR2 and the anti-VEGFR2 nanobody structures. VGFR2 3D structure was modeled using PDB IDs 5T89 with average model confidence of 0.56 ± 0.05. The anti-VEGFR2 nanobody structure was also homology modeled using PDB IDs 5IML with average model confidence of 0.80 ± 0.07. The modeled structures were then refined and validated with the aid of Chiron webserver (https://dokhlab.med.psu.edu/chiron) to ensure all bond lengths, angles and phi/psi angles for all residues are within acceptable range and the backbone of the target proteins are not distorted. The ZDOCK webserver (https://zdock.umassmed.edu) was then used to dock the anti-VEGFR2 nanobody structure over VEGFR2. The complex structure with best ZDOCK score of 1513.881 is presented in Fig. S1. Extracellular domain of VGFR2 (20∼764 aa) composed of 7 immunoglobulin-like subdomains, IgD1 (46∼110 aa), IgD2 (141∼207 aa), IgD3 (224∼320 aa), IgD4 (328∼414 aa), IgD5 (421∼548 aa), IgD6 (551∼660 aa), and IgD7 (667∼753 aa), and the anti-VEGFR2 nanobody binding to the IgD2 and IgD3 of VEGFR-2.


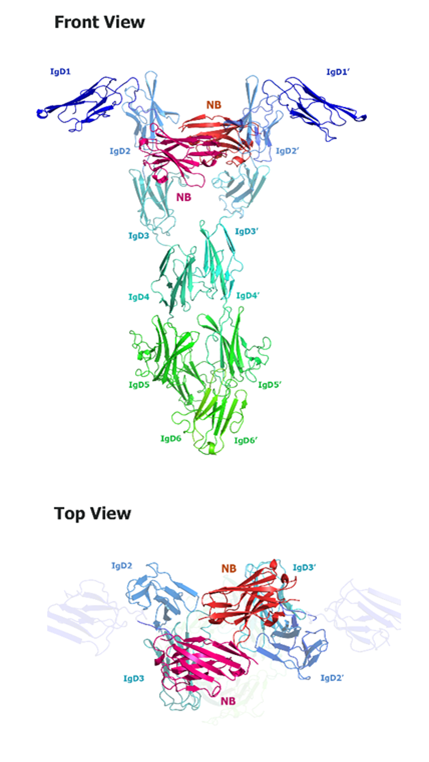


Figure S1: Molecular structure of anti-VEGFR2 nanobody binding to IgD2 and IgD3 of VEGFR-2. The anti-VEGFR2 nanobody binding to the IgD2 and IgD3 of VEGFR-2 (The nanobody can interact the two identical binding sites, either in the front (magenta) or back (orange) view).
